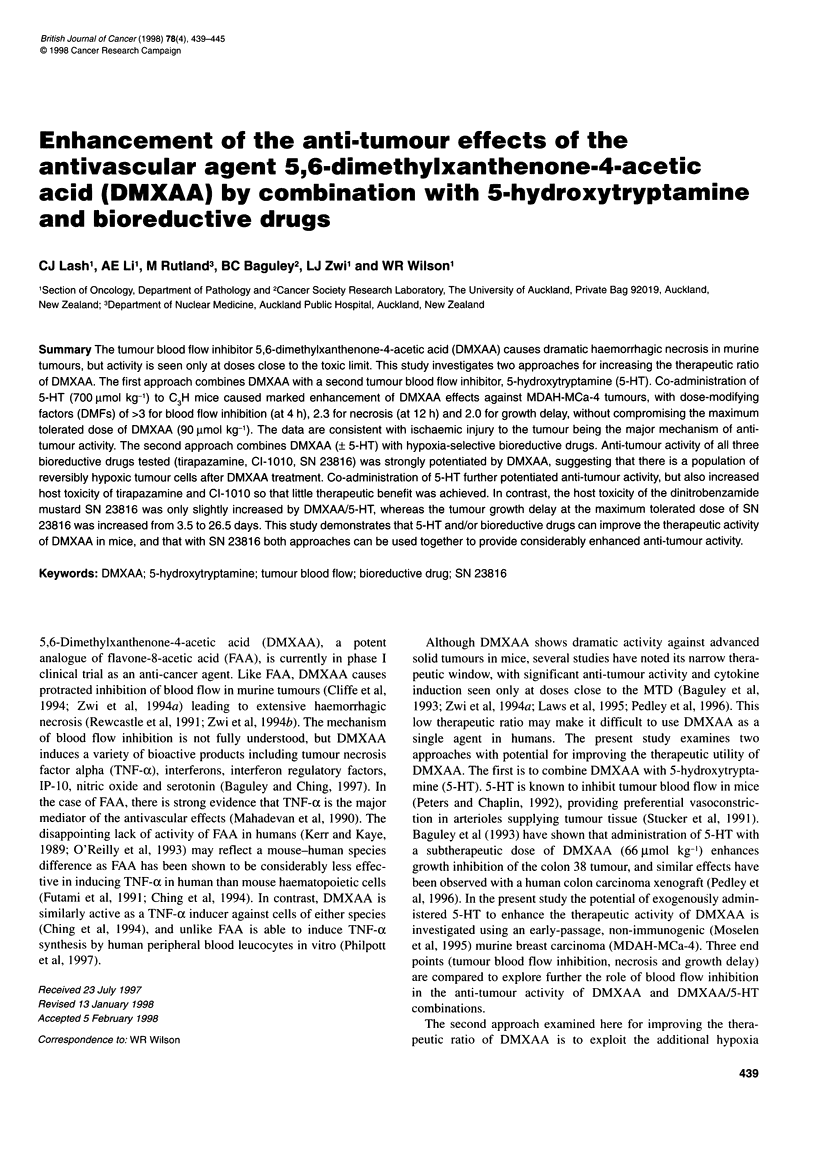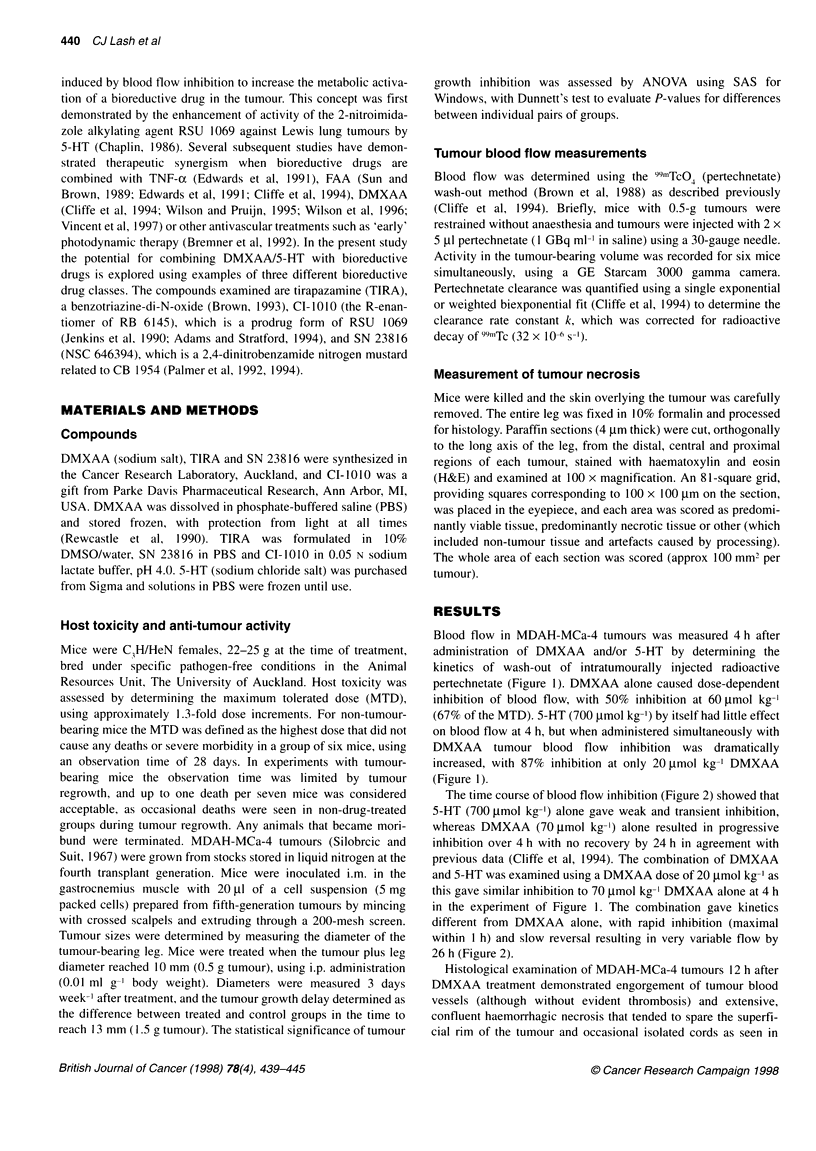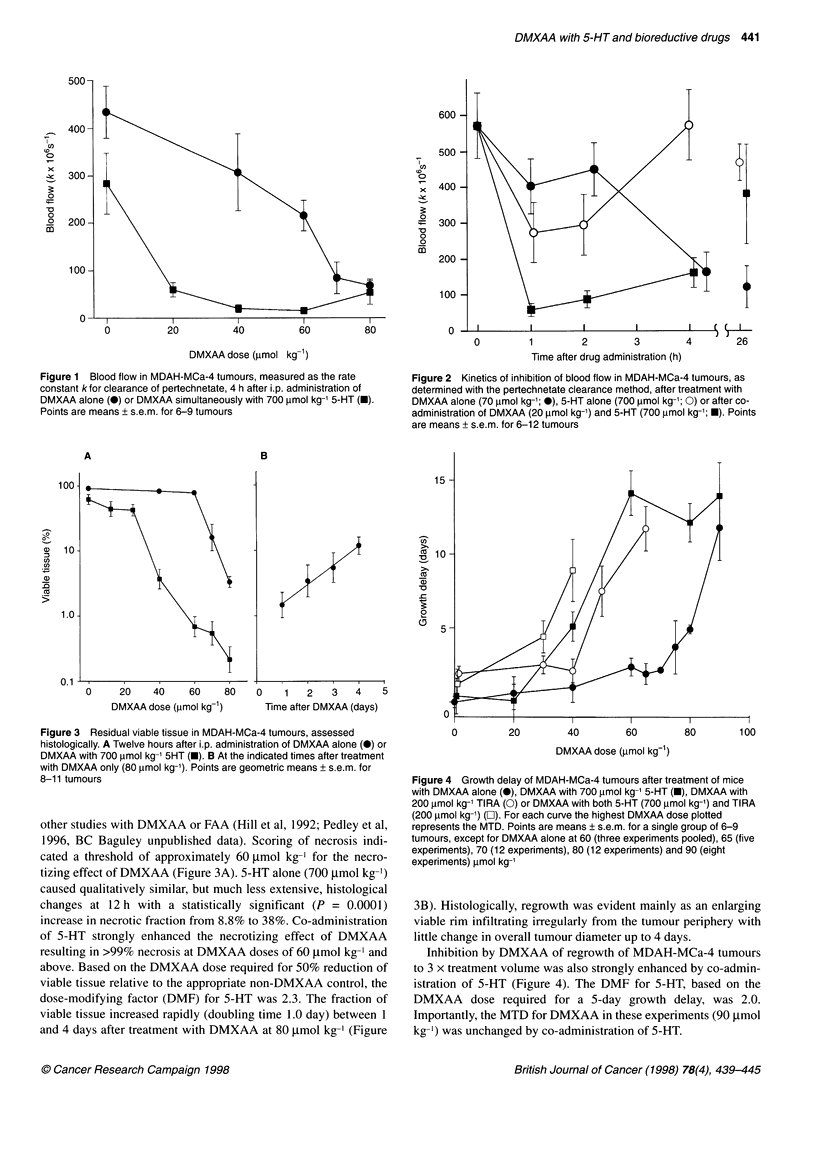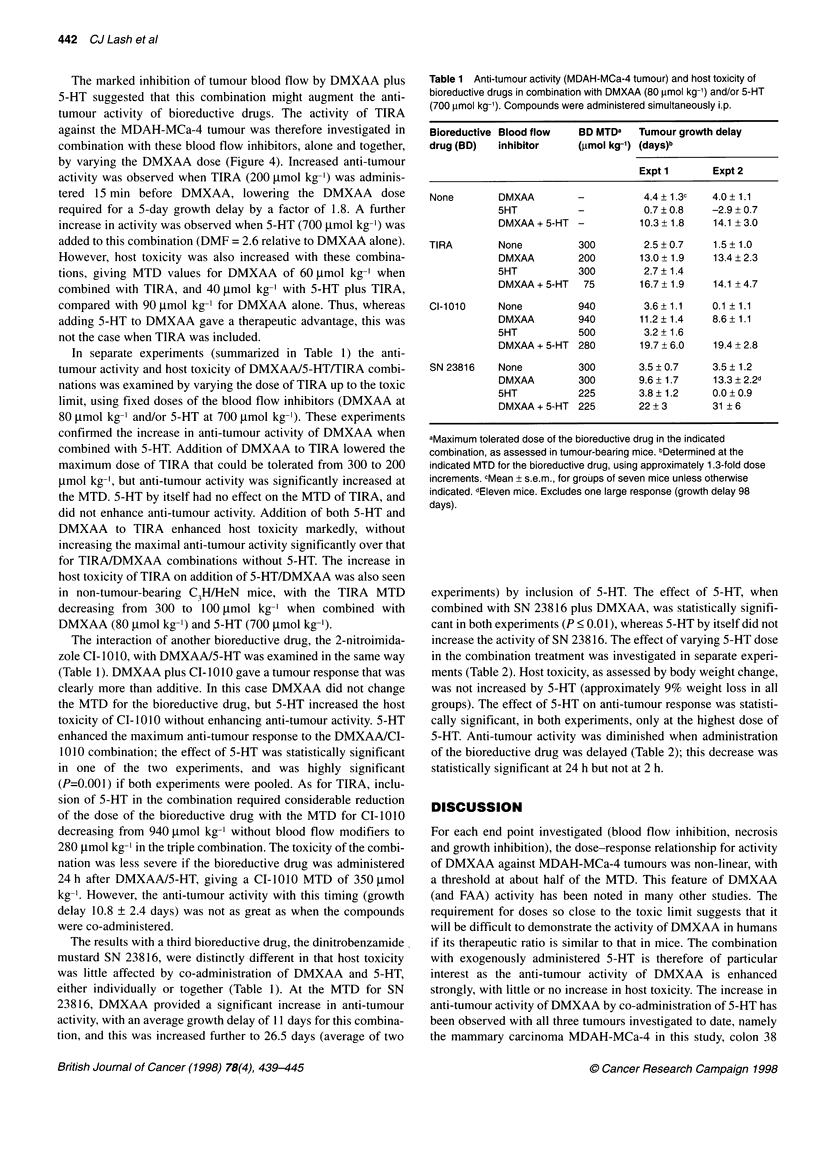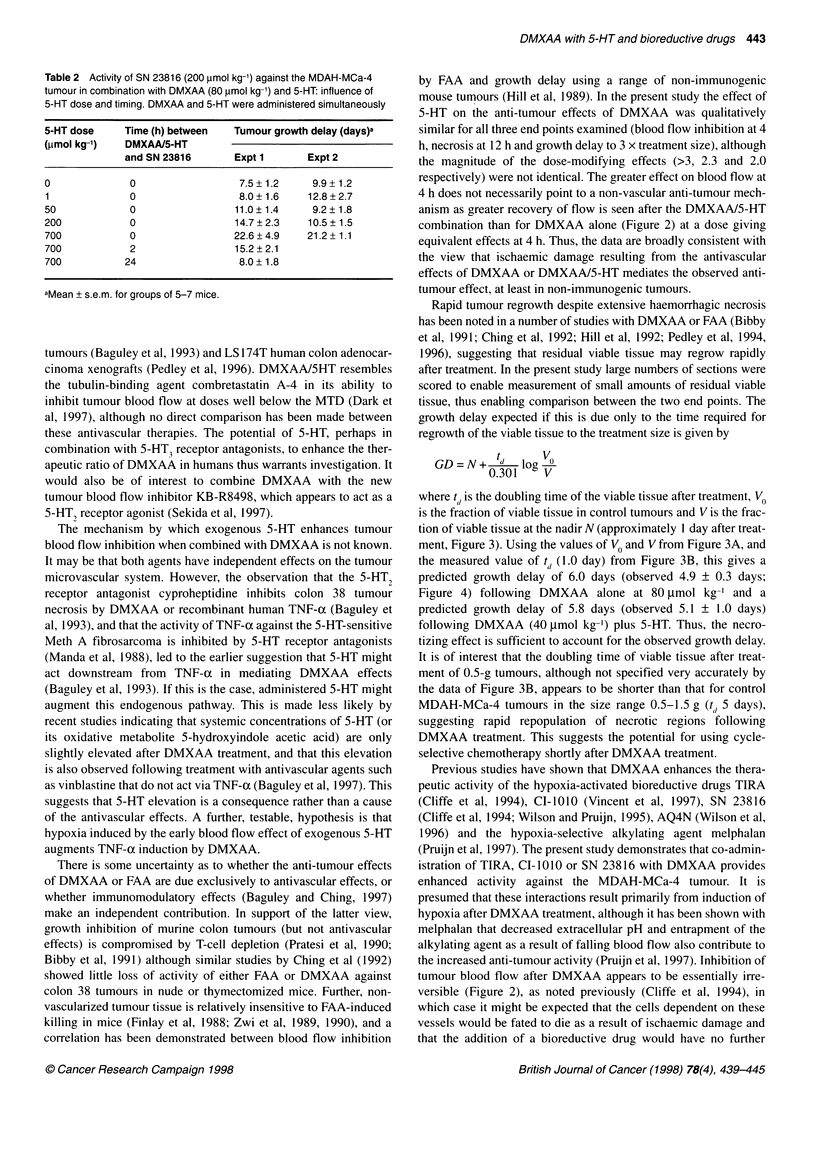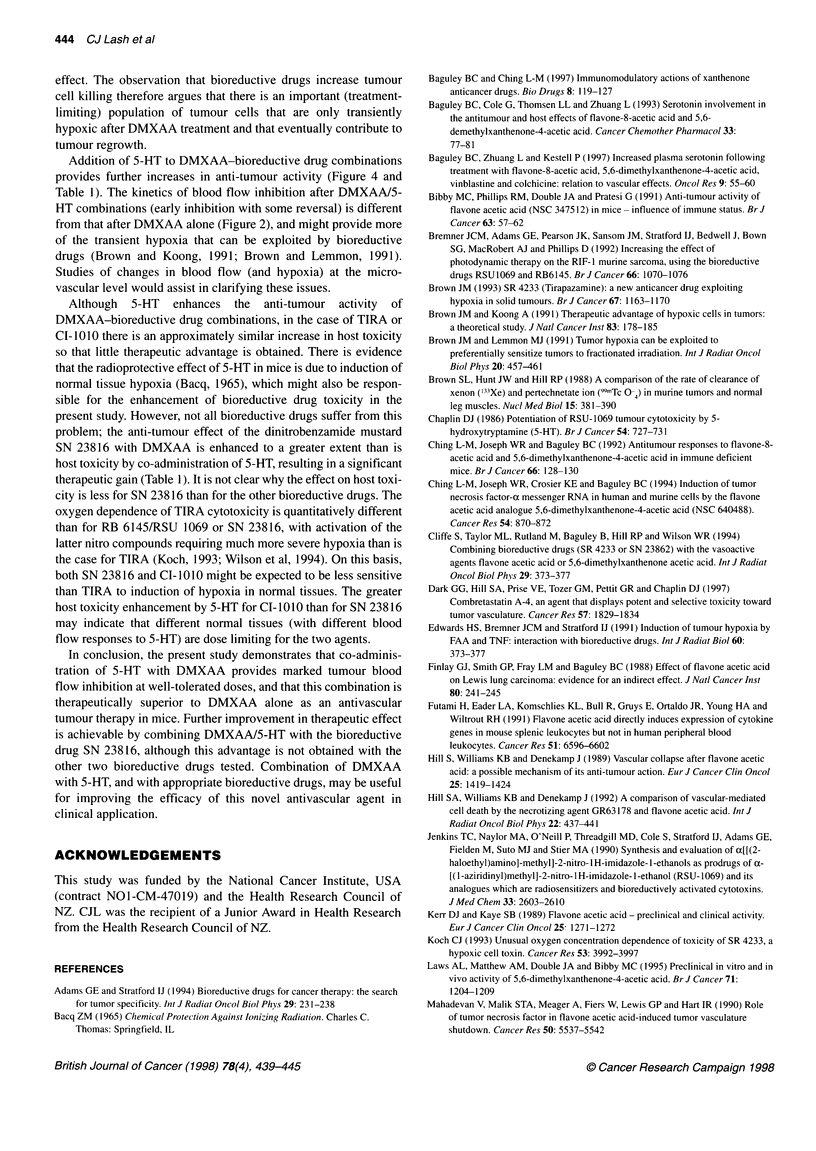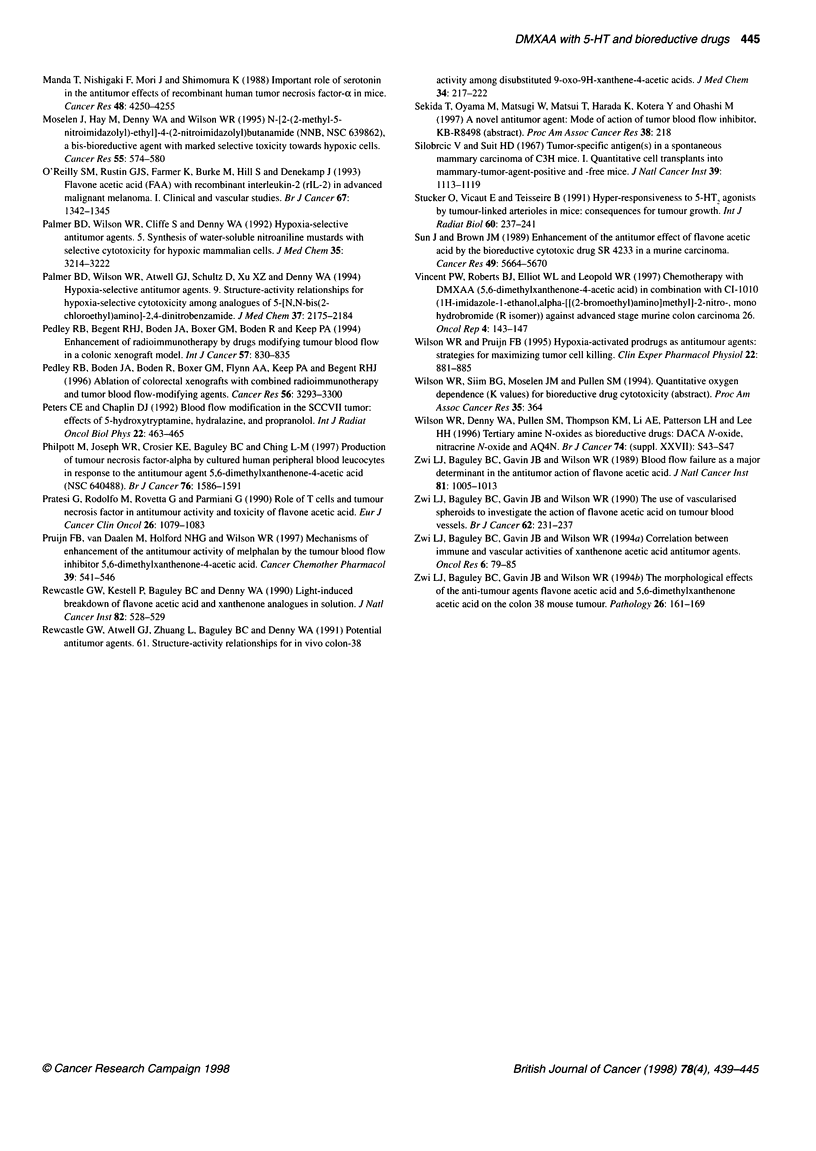# Enhancement of the anti-tumour effects of the antivascular agent 5,6-dimethylxanthenone-4-acetic acid (DMXAA) by combination with 5-hydroxytryptamine and bioreductive drugs.

**DOI:** 10.1038/bjc.1998.512

**Published:** 1998-08

**Authors:** C. J. Lash, A. E. Li, M. Rutland, B. C. Baguley, L. J. Zwi, W. R. Wilson

**Affiliations:** Department of Pathology, The University of Auckland, New Zealand.

## Abstract

The tumour blood flow inhibitor 5,6-dimethylxanthenone-4-acetic acid (DMXAA) causes dramatic haemorrhagic necrosis in murine tumours, but activity is seen only at doses close to the toxic limit. This study investigates two approaches for increasing the therapeutic ratio of DMXAA. The first approach combines DMXAA with a second tumour blood flow inhibitor, 5-hydroxytryptamine (5-HT). Co-administration of 5-HT (700 micromol kg(-1)) to C3H mice caused marked enhancement of DMXAA effects against MDAH-MCa-4 tumours, with dose-modifying factors (DMFs) of >3 for blood flow inhibition (at 4 h), 2.3 for necrosis (at 12 h) and 2.0 for growth delay, without compromising the maximum tolerated dose of DMXAA (90 micromol kg(-1)). The data are consistent with ischaemic injury to the tumour being the major mechanism of anti-tumour activity. The second approach combines DMXAA (+/- 5-HT) with hypoxia-selective bioreductive drugs. Anti-tumour activity of all three bioreductive drugs tested (tirapazamine, CI-1010, SN 23816) was strongly potentiated by DMXAA, suggesting that there is a population of reversibly hypoxic tumour cells after DMXAA treatment. Co-administration of 5-HT further potentiated anti-tumour activity, but also increased host toxicity of tirapazamine and CI-1010 so that little therapeutic benefit was achieved. In contrast, the host toxicity of the dinitrobenzamide mustard SN 23816 was only slightly increased by DMXAA/5-HT, whereas the tumour growth delay at the maximum tolerated dose of SN 23816 was increased from 3.5 to 26.5 days. This study demonstrates that 5-HT and/or bioreductive drugs can improve the therapeutic activity of DMXAA in mice, and that with SN 23816 both approaches can be used together to provide considerably enhanced anti-tumour activity.


British Journal of Cancer (1998) 78(4), 439–445
© 1998 Cancer Research Campaign

### Enhancement of the anti-tumour effects of the antivascular agent 5,6-dimethylxanthenone-4-acetic acid (DMXAA) by combination with 5-hydroxytryptamine and bioreductive drugs

**CJ Lash[1], AE Li[1], M Rutland[3], BC Baguley[2], LJ Zwi[1] and WR Wilson[1]**

[1]Section of Oncology, Department of Pathology and [2]Cancer Society Research Laboratory, The University of Auckland, Private Bag 92019, Auckland, New Zealand; [3]Department of Nuclear Medicine, Auckland Public Hospital, Auckland, New Zealand

**Summary** The tumour blood flow inhibitor 5,6-dimethylxanthenone-4-acetic acid (DMXAA) causes dramatic haemorrhagic necrosis in murine tumours, but activity is seen only at doses close to the toxic limit. This study investigates two approaches for increasing the therapeutic ratio of DMXAA. The first approach combines DMXAA with a second tumour blood flow inhibitor, 5-hydroxytryptamine (5-HT). Co-administration of 5-HT (700 μmol $kg^{-1}$) to $C_3H$ mice caused marked enhancement of DMXAA effects against MDAH-MCa-4 tumours, with dose-modifying factors (DMFs) of >3 for blood flow inhibition (at 4 h), 2.3 for necrosis (at 12 h) and 2.0 for growth delay, without compromising the maximum tolerated dose of DMXAA (90 μmol $kg^{-1}$). The data are consistent with ischaemic injury to the tumour being the major mechanism of anti-tumour activity. The second approach combines DMXAA (± 5-HT) with hypoxia-selective bioreductive drugs. Anti-tumour activity of all three bioreductive drugs tested (tirapazamine, CI-1010, SN 23816) was strongly potentiated by DMXAA, suggesting that there is a population of reversibly hypoxic tumour cells after DMXAA treatment. Co-administration of 5-HT further potentiated anti-tumour activity, but also increased host toxicity of tirapazamine and CI-1010 so that little therapeutic benefit was achieved. In contrast, the host toxicity of the dinitrobenzamide mustard SN 23816 was only slightly increased by DMXAA/5-HT, whereas the tumour growth delay at the maximum tolerated dose of SN 23816 was increased from 3.5 to 26.5 days. This study demonstrates that 5-HT and/or bioreductive drugs can improve the therapeutic activity of DMXAA in mice, and that with SN 23816 both approaches can be used together to provide considerably enhanced anti-tumour activity.

**Keywords:** DMXAA; 5-hydroxytryptamine; tumour blood flow; bioreductive drug; SN 23816

5,6-Dimethylxanthenone-4-acetic acid (DMXAA), a potent analogue of flavone-8-acetic acid (FAA), is currently in phase I clinical trial as an anti-cancer agent. Like FAA, DMXAA causes protracted inhibition of blood flow in murine tumours (Cliffe et al, 1994; Zwi et al, 1994*a*) leading to extensive haemorrhagic necrosis (Rewcastle et al, 1991; Zwi et al, 1994*b*). The mechanism of blood flow inhibition is not fully understood, but DMXAA induces a variety of bioactive products including tumour necrosis factor alpha (TNF-α), interferons, interferon regulatory factors, IP-10, nitric oxide and serotonin (Baguley and Ching, 1997). In the case of FAA, there is strong evidence that TNF-α is the major mediator of the antivascular effects (Mahadevan et al, 1990). The disappointing lack of activity of FAA in humans (Kerr and Kaye, 1989; O'Reilly et al, 1993) may reflect a mouse–human species difference as FAA has been shown to be considerably less effective in inducing TNF-α in human than mouse haematopoietic cells (Futami et al, 1991; Ching et al, 1994). In contrast, DMXAA is similarly active as a TNF-α inducer against cells of either species (Ching et al, 1994), and unlike FAA is able to induce TNF-α synthesis by human peripheral blood leucocytes in vitro (Philpott et al, 1997).

*Received 23 July 1997*
*Revised 13 January 1998*
*Accepted 5 February 1998*

*Correspondence to:* WR Wilson

Although DMXAA shows dramatic activity against advanced solid tumours in mice, several studies have noted its narrow therapeutic window, with significant anti-tumour activity and cytokine induction seen only at doses close to the MTD (Baguley et al, 1993; Zwi et al, 1994*a*; Laws et al, 1995; Pedley et al, 1996). This low therapeutic ratio may make it difficult to use DMXAA as a single agent in humans. The present study examines two approaches with potential for improving the therapeutic utility of DMXAA. The first is to combine DMXAA with 5-hydroxytryptamine (5-HT). 5-HT is known to inhibit tumour blood flow in mice (Peters and Chaplin, 1992), providing preferential vasoconstriction in arterioles supplying tumour tissue (Stucker et al, 1991). Baguley et al (1993) have shown that administration of 5-HT with a subtherapeutic dose of DMXAA (66 μmol $kg^{-1}$) enhances growth inhibition of the colon 38 tumour, and similar effects have been observed with a human colon carcinoma xenograft (Pedley et al, 1996). In the present study the potential of exogenously administered 5-HT to enhance the therapeutic activity of DMXAA is investigated using an early-passage, non-immunogenic (Moselen et al, 1995) murine breast carcinoma (MDAH-MCa-4). Three end points (tumour blood flow inhibition, necrosis and growth delay) are compared to explore further the role of blood flow inhibition in the anti-tumour activity of DMXAA and DMXAA/5-HT combinations.

The second approach examined here for improving the therapeutic ratio of DMXAA is to exploit the additional hypoxia induced by blood flow inhibition to increase the metabolic activation of a bioreductive drug in the tumour. This concept was first demonstrated by the enhancement of activity of the 2-nitroimidazole alkylating agent RSU 1069 against Lewis lung tumours by 5-HT (Chaplin, 1986). Several subsequent studies have demonstrated therapeutic synergism when bioreductive drugs are combined with TNF-α (Edwards et al, 1991), FAA (Sun and Brown, 1989; Edwards et al, 1991; Cliffe et al, 1994), DMXAA (Cliffe et al, 1994; Wilson and Pruijn, 1995; Wilson et al, 1996; Vincent et al, 1997) or other antivascular treatments such as 'early' photodynamic therapy (Bremner et al, 1992). In the present study the potential for combining DMXAA/5-HT with bioreductive drugs is explored using examples of three different bioreductive drug classes. The compounds examined are tirapazamine (TIRA), a benzotriazine-di-N-oxide (Brown, 1993), CI-1010 (the R-enantiomer of RB 6145), which is a prodrug form of RSU 1069 (Jenkins et al, 1990; Adams and Stratford, 1994), and SN 23816 (NSC 646394), which is a 2,4-dinitrobenzamide nitrogen mustard related to CB 1954 (Palmer et al, 1992, 1994).

### MATERIALS AND METHODS

### Compounds

DMXAA (sodium salt), TIRA and SN 23816 were synthesized in the Cancer Research Laboratory, Auckland, and CI-1010 was a gift from Parke Davis Pharmaceutical Research, Ann Arbor, MI, USA. DMXAA was dissolved in phosphate-buffered saline (PBS) and stored frozen, with protection from light at all times (Rewcastle et al, 1990). TIRA was formulated in 10% DMSO/water, SN 23816 in PBS and CI-1010 in 0.05 N sodium lactate buffer, pH 4.0. 5-HT (sodium chloride salt) was purchased from Sigma and solutions in PBS were frozen until use.

### Host toxicity and anti-tumour activity

Mice were $C_3H$/HeN females, 22–25 g at the time of treatment, bred under specific pathogen-free conditions in the Animal Resources Unit, The University of Auckland. Host toxicity was assessed by determining the maximum tolerated dose (MTD), using approximately 1.3-fold dose increments. For non-tumour-bearing mice the MTD was defined as the highest dose that did not cause any deaths or severe morbidity in a group of six mice, using an observation time of 28 days. In experiments with tumour-bearing mice the observation time was limited by tumour regrowth, and up to one death per seven mice was considered acceptable, as occasional deaths were seen in non-drug-treated groups during tumour regrowth. Any animals that became moribund were terminated. MDAH-MCa-4 tumours (Silobrcic and Suit, 1967) were grown from stocks stored in liquid nitrogen at the fourth transplant generation. Mice were inoculated i.m. in the gastrocnemius muscle with 20 μl of a cell suspension (5 mg packed cells) prepared from fifth-generation tumours by mincing with crossed scalpels and extruding through a 200-mesh screen. Tumour sizes were determined by measuring the diameter of the tumour-bearing leg. Mice were treated when the tumour plus leg diameter reached 10 mm (0.5 g tumour), using i.p. administration (0.01 ml $g^{-1}$ body weight). Diameters were measured 3 days $week^{-1}$ after treatment, and the tumour growth delay determined as the difference between treated and control groups in the time to reach 13 mm (1.5 g tumour). The statistical significance of tumour growth inhibition was assessed by ANOVA using SAS for Windows, with Dunnett's test to evaluate *P*-values for differences between individual pairs of groups.

### Tumour blood flow measurements

Blood flow was determined using the $^{99m}TcO_4$ (pertechnetate) wash-out method (Brown et al, 1988) as described previously (Cliffe et al, 1994). Briefly, mice with 0.5-g tumours were restrained without anaesthesia and tumours were injected with 2 × 5 μl pertechnetate (1 GBq $ml^{-1}$ in saline) using a 30-gauge needle. Activity in the tumour-bearing volume was recorded for six mice simultaneously, using a GE Starcam 3000 gamma camera. Pertechnetate clearance was quantified using a single exponential or weighted biexponential fit (Cliffe et al, 1994) to determine the clearance rate constant *k*, which was corrected for radioactive decay of $^{99m}$Tc ($32 \times 10^{-6}$ $s^{-1}$).

### Measurement of tumour necrosis

Mice were killed and the skin overlying the tumour was carefully removed. The entire leg was fixed in 10% formalin and processed for histology. Paraffin sections (4 μm thick) were cut, orthogonally to the long axis of the leg, from the distal, central and proximal regions of each tumour, stained with haematoxylin and eosin (H&E) and examined at 100 × magnification. An 81-square grid, providing squares corresponding to 100 × 100 μm on the section, was placed in the eyepiece, and each area was scored as predominantly viable tissue, predominantly necrotic tissue or other (which included non-tumour tissue and artefacts caused by processing). The whole area of each section was scored (approx 100 $mm^2$ per tumour).

### RESULTS

Blood flow in MDAH-MCa-4 tumours was measured 4 h after administration of DMXAA and/or 5-HT by determining the kinetics of wash-out of intratumourally injected radioactive pertechnetate (Figure 1). DMXAA alone caused dose-dependent inhibition of blood flow, with 50% inhibition at 60 μmol $kg^{-1}$ (67% of the MTD). 5-HT (700 μmol $kg^{-1}$) by itself had little effect on blood flow at 4 h, but when administered simultaneously with DMXAA tumour blood flow inhibition was dramatically increased, with 87% inhibition at only 20 μmol $kg^{-1}$ DMXAA (Figure 1).

The time course of blood flow inhibition (Figure 2) showed that 5-HT (700 μmol $kg^{-1}$) alone gave weak and transient inhibition, whereas DMXAA (70 μmol $kg^{-1}$) alone resulted in progressive inhibition over 4 h with no recovery by 24 h in agreement with previous data (Cliffe et al, 1994). The combination of DMXAA and 5-HT was examined using a DMXAA dose of 20 μmol $kg^{-1}$ as this gave similar inhibition to 70 μmol $kg^{-1}$ DMXAA alone at 4 h in the experiment of Figure 1. The combination gave kinetics different from DMXAA alone, with rapid inhibition (maximal within 1 h) and slow reversal resulting in very variable flow by 26 h (Figure 2).

Histological examination of MDAH-MCa-4 tumours 12 h after DMXAA treatment demonstrated engorgement of tumour blood vessels (although without evident thrombosis) and extensive, confluent haemorrhagic necrosis that tended to spare the superficial rim of the tumour and occasional isolated cords as seen in

 © Cancer Research Campaign 1998

Figure 1 Blood flow in MDAH-MCa-4 tumours, measured as the rate constant *k* for clearance of pertechnetate, 4 h after i.p. administration of DMXAA alone (●) or DMXAA simultaneously with 700 μmol kg$^{-1}$ 5-HT (■). Points are means ± s.e.m. for 6–9 tumours

Figure 2 Kinetics of inhibition of blood flow in MDAH-MCa-4 tumours, as determined with the pertechnetate clearance method, after treatment with DMXAA alone (70 μmol kg$^{-1}$; ●), 5-HT alone (700 μmol kg$^{-1}$; ○) or after co-administration of DMXAA (20 μmol kg$^{-1}$) and 5-HT (700 μmol kg$^{-1}$; ■). Points are means ± s.e.m. for 6–12 tumours

Figure 3 Residual viable tissue in MDAH-MCa-4 tumours, assessed histologically. **A** Twelve hours after i.p. administration of DMXAA alone (●) or DMXAA with 700 μmol kg$^{-1}$ 5HT (■). **B** At the indicated times after treatment with DMXAA only (80 μmol kg$^{-1}$). Points are geometric means ± s.e.m. for 8–11 tumours

Figure 4 Growth delay of MDAH-MCa-4 tumours after treatment of mice with DMXAA alone (●), DMXAA with 700 μmol kg$^{-1}$ 5-HT (■), DMXAA with 200 μmol kg$^{-1}$ TIRA (○) or DMXAA with both 5-HT (700 μmol kg$^{-1}$) and TIRA (200 μmol kg$^{-1}$) (□). For each curve the highest DMXAA dose plotted represents the MTD. Points are means ± s.e.m. for a single group of 6–9 tumours, except for DMXAA alone at 60 (three experiments pooled), 65 (five experiments), 70 (12 experiments), 80 (12 experiments) and 90 (eight experiments) μmol kg$^{-1}$

other studies with DMXAA or FAA (Hill et al, 1992; Pedley et al, 1996, BC Baguley unpublished data). Scoring of necrosis indicated a threshold of approximately 60 μmol kg$^{-1}$ for the necrotizing effect of DMXAA (Figure 3A). 5-HT alone (700 μmol kg$^{-1}$) caused qualitatively similar, but much less extensive, histological changes at 12 h with a statistically significant ($P$ = 0.0001) increase in necrotic fraction from 8.8% to 38%. Co-administration of 5-HT strongly enhanced the necrotizing effect of DMXAA resulting in >99% necrosis at DMXAA doses of 60 μmol kg$^{-1}$ and above. Based on the DMXAA dose required for 50% reduction of viable tissue relative to the appropriate non-DMXAA control, the dose-modifying factor (DMF) for 5-HT was 2.3. The fraction of viable tissue increased rapidly (doubling time 1.0 day) between 1 and 4 days after treatment with DMXAA at 80 μmol kg$^{-1}$ (Figure 3B). Histologically, regrowth was evident mainly as an enlarging viable rim infiltrating irregularly from the tumour periphery with little change in overall tumour diameter up to 4 days.

Inhibition by DMXAA of regrowth of MDAH-MCa-4 tumours to 3 × treatment volume was also strongly enhanced by co-administration of 5-HT (Figure 4). The DMF for 5-HT, based on the DMXAA dose required for a 5-day growth delay, was 2.0. Importantly, the MTD for DMXAA in these experiments (90 μmol kg$^{-1}$) was unchanged by co-administration of 5-HT.

© Cancer Research Campaign 1998

The marked inhibition of tumour blood flow by DMXAA plus 5-HT suggested that this combination might augment the anti-tumour activity of bioreductive drugs. The activity of TIRA against the MDAH-MCa-4 tumour was therefore investigated in combination with these blood flow inhibitors, alone and together, by varying the DMXAA dose (Figure 4). Increased anti-tumour activity was observed when TIRA (200 μmol $kg^{-1}$) was administered 15 min before DMXAA, lowering the DMXAA dose required for a 5-day growth delay by a factor of 1.8. A further increase in activity was observed when 5-HT (700 μmol $kg^{-1}$) was added to this combination (DMF = 2.6 relative to DMXAA alone). However, host toxicity was also increased with these combinations, giving MTD values for DMXAA of 60 μmol $kg^{-1}$ when combined with TIRA, and 40 μmol $kg^{-1}$ with 5-HT plus TIRA, compared with 90 μmol $kg^{-1}$ for DMXAA alone. Thus, whereas adding 5-HT to DMXAA gave a therapeutic advantage, this was not the case when TIRA was included.

In separate experiments (summarized in Table 1) the anti-tumour activity and host toxicity of DMXAA/5-HT/TIRA combinations was examined by varying the dose of TIRA up to the toxic limit, using fixed doses of the blood flow inhibitors (DMXAA at 80 μmol $kg^{-1}$ and/or 5-HT at 700 μmol $kg^{-1}$). These experiments confirmed the increase in anti-tumour activity of DMXAA when combined with 5-HT. Addition of DMXAA to TIRA lowered the maximum dose of TIRA that could be tolerated from 300 to 200 μmol $kg^{-1}$, but anti-tumour activity was significantly increased at the MTD. 5-HT by itself had no effect on the MTD of TIRA, and did not enhance anti-tumour activity. Addition of both 5-HT and DMXAA to TIRA enhanced host toxicity markedly, without increasing the maximal anti-tumour activity significantly over that for TIRA/DMXAA combinations without 5-HT. The increase in host toxicity of TIRA on addition of 5-HT/DMXAA was also seen in non-tumour-bearing $C_3H$/HeN mice, with the TIRA MTD decreasing from 300 to 100 μmol $kg^{-1}$ when combined with DMXAA (80 μmol $kg^{-1}$) and 5-HT (700 μmol $kg^{-1}$).

The interaction of another bioreductive drug, the 2-nitroimidazole CI-1010, with DMXAA/5-HT was examined in the same way (Table 1). DMXAA plus CI-1010 gave a tumour response that was clearly more than additive. In this case DMXAA did not change the MTD for the bioreductive drug, but 5-HT increased the host toxicity of CI-1010 without enhancing anti-tumour activity. 5-HT enhanced the maximum anti-tumour response to the DMXAA/CI-1010 combination; the effect of 5-HT was statistically significant in one of the two experiments, and was highly significant ($P$=0.001) if both experiments were pooled. As for TIRA, inclusion of 5-HT in the combination required considerable reduction of the dose of the bioreductive drug with the MTD for CI-1010 decreasing from 940 μmol $kg^{-1}$ without blood flow modifiers to 280 μmol $kg^{-1}$ in the triple combination. The toxicity of the combination was less severe if the bioreductive drug was administered 24 h after DMXAA/5-HT, giving a CI-1010 MTD of 350 μmol $kg^{-1}$. However, the anti-tumour activity with this timing (growth delay 10.8 ± 2.4 days) was not as great as when the compounds were co-administered.

The results with a third bioreductive drug, the dinitrobenzamide mustard SN 23816, were distinctly different in that host toxicity was little affected by co-administration of DMXAA and 5-HT, either individually or together (Table 1). At the MTD for SN 23816, DMXAA provided a significant increase in anti-tumour activity, with an average growth delay of 11 days for this combination, and this was increased further to 26.5 days (average of two experiments) by inclusion of 5-HT. The effect of 5-HT, when combined with SN 23816 plus DMXAA, was statistically significant in both experiments ($P \leq 0.01$), whereas 5-HT by itself did not increase the activity of SN 23816. The effect of varying 5-HT dose in the combination treatment was investigated in separate experiments (Table 2). Host toxicity, as assessed by body weight change, was not increased by 5-HT (approximately 9% weight loss in all groups). The effect of 5-HT on anti-tumour response was statistically significant, in both experiments, only at the highest dose of 5-HT. Anti-tumour activity was diminished when administration of the bioreductive drug was delayed (Table 2); this decrease was statistically significant at 24 h but not at 2 h.

**Table 1** Anti-tumour activity (MDAH-MCa-4 tumour) and host toxicity of bioreductive drugs in combination with DMXAA (80 μmol $kg^{-1}$) and/or 5-HT (700 μmol $kg^{-1}$). Compounds were administered simultaneously i.p.

| Bioreductive drug (BD) | Blood flow inhibitor | BD MTD[a] (μmol $kg^{-1}$) | Tumour growth delay (days)[b] Expt 1 | Expt 2 |
|---|---|---|---|---|
| None | DMXAA | – | 4.4 ± 1.3[c] | 4.0 ± 1.1 |
| | 5HT | – | 0.7 ± 0.8 | –2.9 ± 0.7 |
| | DMXAA + 5-HT | – | 10.3 ± 1.8 | 14.1 ± 3.0 |
| TIRA | None | 300 | 2.5 ± 0.7 | 1.5 ± 1.0 |
| | DMXAA | 200 | 13.0 ± 1.9 | 13.4 ± 2.3 |
| | 5HT | 300 | 2.7 ± 1.4 | |
| | DMXAA + 5-HT | 75 | 16.7 ± 1.9 | 14.1 ± 4.7 |
| CI-1010 | None | 940 | 3.6 ± 1.1 | 0.1 ± 1.1 |
| | DMXAA | 940 | 11.2 ± 1.4 | 8.6 ± 1.1 |
| | 5HT | 500 | 3.2 ± 1.6 | |
| | DMXAA + 5-HT | 280 | 19.7 ± 6.0 | 19.4 ± 2.8 |
| SN 23816 | None | 300 | 3.5 ± 0.7 | 3.5 ± 1.2 |
| | DMXAA | 300 | 9.6 ± 1.7 | 13.3 ± 2.2[d] |
| | 5HT | 225 | 3.8 ± 1.2 | 0.0 ± 0.9 |
| | DMXAA + 5-HT | 225 | 22 ± 3 | 31 ± 6 |

[a]Maximum tolerated dose of the bioreductive drug in the indicated combination, as assessed in tumour-bearing mice. [b]Determined at the indicated MTD for the bioreductive drug, using approximately 1.3-fold dose increments. [c]Mean ± s.e.m., for groups of seven mice unless otherwise indicated. [d]Eleven mice. Excludes one large response (growth delay 98 days).

### DISCUSSION

For each end point investigated (blood flow inhibition, necrosis and growth inhibition), the dose–response relationship for activity of DMXAA against MDAH-MCa-4 tumours was non-linear, with a threshold at about half of the MTD. This feature of DMXAA (and FAA) activity has been noted in many other studies. The requirement for doses so close to the toxic limit suggests that it will be difficult to demonstrate the activity of DMXAA in humans if its therapeutic ratio is similar to that in mice. The combination with exogenously administered 5-HT is therefore of particular interest as the anti-tumour activity of DMXAA is enhanced strongly, with little or no increase in host toxicity. The increase in anti-tumour activity of DMXAA by co-administration of 5-HT has been observed with all three tumours investigated to date, namely the mammary carcinoma MDAH-MCa-4 in this study, colon 38

© Cancer Research Campaign 1998

**Table 2** Activity of SN 23816 (200 μmol kg$^{-1}$) against the MDAH-MCa-4 tumour in combination with DMXAA (80 μmol kg$^{-1}$) and 5-HT: influence of 5-HT dose and timing. DMXAA and 5-HT were administered simultaneously

| 5-HT dose (μmol kg$^{-1}$) | Time (h) between DMXAA/5-HT and SN 23816 | Tumour growth delay (days)[a] | |
|---|---|---|---|
| | | Expt 1 | Expt 2 |
| 0 | 0 | 7.5 ± 1.2 | 9.9 ± 1.2 |
| 1 | 0 | 8.0 ± 1.6 | 12.8 ± 2.7 |
| 50 | 0 | 11.0 ± 1.4 | 9.2 ± 1.8 |
| 200 | 0 | 14.7 ± 2.3 | 10.5 ± 1.5 |
| 700 | 0 | 22.6 ± 4.9 | 21.2 ± 1.1 |
| 700 | 2 | 15.2 ± 2.1 | |
| 700 | 24 | 8.0 ± 1.8 | |

[a]Mean ± s.e.m. for groups of 5–7 mice.

tumours (Baguley et al, 1993) and LS174T human colon adenocarcinoma xenografts (Pedley et al, 1996). DMXAA/5HT resembles the tubulin-binding agent combretastatin A-4 in its ability to inhibit tumour blood flow at doses well below the MTD (Dark et al, 1997), although no direct comparison has been made between these antivascular therapies. The potential of 5-HT, perhaps in combination with 5-$HT_3$ receptor antagonists, to enhance the therapeutic ratio of DMXAA in humans thus warrants investigation. It would also be of interest to combine DMXAA with the new tumour blood flow inhibitor KB-R8498, which appears to act as a 5-$HT_2$ receptor agonist (Sekida et al, 1997).

The mechanism by which exogenous 5-HT enhances tumour blood flow inhibition when combined with DMXAA is not known. It may be that both agents have independent effects on the tumour microvascular system. However, the observation that the 5-$HT_2$ receptor antagonist cyproheptidine inhibits colon 38 tumour necrosis by DMXAA or recombinant human TNF-α (Baguley et al, 1993), and that the activity of TNF-α against the 5-HT-sensitive Meth A fibrosarcoma is inhibited by 5-HT receptor antagonists (Manda et al, 1988), led to the earlier suggestion that 5-HT might act downstream from TNF-α in mediating DMXAA effects (Baguley et al, 1993). If this is the case, administered 5-HT might augment this endogenous pathway. This is made less likely by recent studies indicating that systemic concentrations of 5-HT (or its oxidative metabolite 5-hydroxyindole acetic acid) are only slightly elevated after DMXAA treatment, and that this elevation is also observed following treatment with antivascular agents such as vinblastine that do not act via TNF-α (Baguley et al, 1997). This suggests that 5-HT elevation is a consequence rather than a cause of the antivascular effects. A further, testable, hypothesis is that hypoxia induced by the early blood flow effect of exogenous 5-HT augments TNF-α induction by DMXAA.

There is some uncertainty as to whether the anti-tumour effects of DMXAA or FAA are due exclusively to antivascular effects, or whether immunomodulatory effects (Baguley and Ching, 1997) make an independent contribution. In support of the latter view, growth inhibition of murine colon tumours (but not antivascular effects) is compromised by T-cell depletion (Pratesi et al, 1990; Bibby et al, 1991) although similar studies by Ching et al (1992) showed little loss of activity of either FAA or DMXAA against colon 38 tumours in nude or thymectomized mice. Further, non-vascularized tumour tissue is relatively insensitive to FAA-induced killing in mice (Finlay et al, 1988; Zwi et al, 1989, 1990), and a correlation has been demonstrated between blood flow inhibition by FAA and growth delay using a range of non-immunogenic mouse tumours (Hill et al, 1989). In the present study the effect of 5-HT on the anti-tumour effects of DMXAA was qualitatively similar for all three end points examined (blood flow inhibition at 4 h, necrosis at 12 h and growth delay to 3 × treatment size), although the magnitude of the dose-modifying effects (>3, 2.3 and 2.0 respectively) were not identical. The greater effect on blood flow at 4 h does not necessarily point to a non-vascular anti-tumour mechanism as greater recovery of flow is seen after the DMXAA/5-HT combination than for DMXAA alone (Figure 2) at a dose giving equivalent effects at 4 h. Thus, the data are broadly consistent with the view that ischaemic damage resulting from the antivascular effects of DMXAA or DMXAA/5-HT mediates the observed anti-tumour effect, at least in non-immunogenic tumours.

Rapid tumour regrowth despite extensive haemorrhagic necrosis has been noted in a number of studies with DMXAA or FAA (Bibby et al, 1991; Ching et al, 1992; Hill et al, 1992; Pedley et al, 1994, 1996), suggesting that residual viable tissue may regrow rapidly after treatment. In the present study large numbers of sections were scored to enable measurement of small amounts of residual viable tissue, thus enabling comparison between the two end points. The growth delay expected if this is due only to the time required for regrowth of the viable tissue to the treatment size is given by

$$GD = N + \frac{t_d}{0.301} \log \frac{V_0}{V}$$

where $t_d$ is the doubling time of the viable tissue after treatment, $V_0$ is the fraction of viable tissue in control tumours and $V$ is the fraction of viable tissue at the nadir $N$ (approximately 1 day after treatment, Figure 3). Using the values of $V_0$ and $V$ from Figure 3A, and the measured value of $t_d$ (1.0 day) from Figure 3B, this gives a predicted growth delay of 6.0 days (observed 4.9 ± 0.3 days; Figure 4) following DMXAA alone at 80 μmol kg$^{-1}$ and a predicted growth delay of 5.8 days (observed 5.1 ± 1.0 days) following DMXAA (40 μmol kg$^{-1}$) plus 5-HT. Thus, the necrotizing effect is sufficient to account for the observed growth delay. It is of interest that the doubling time of viable tissue after treatment of 0.5-g tumours, although not specified very accurately by the data of Figure 3B, appears to be shorter than that for control MDAH-MCa-4 tumours in the size range 0.5–1.5 g ($t_d$ 5 days), suggesting rapid repopulation of necrotic regions following DMXAA treatment. This suggests the potential for using cycle-selective chemotherapy shortly after DMXAA treatment.

Previous studies have shown that DMXAA enhances the therapeutic activity of the hypoxia-activated bioreductive drugs TIRA (Cliffe et al, 1994), CI-1010 (Vincent et al, 1997), SN 23816 (Cliffe et al, 1994; Wilson and Pruijn, 1995), AQ4N (Wilson et al, 1996) and the hypoxia-selective alkylating agent melphalan (Pruijn et al, 1997). The present study demonstrates that co-administration of TIRA, CI-1010 or SN 23816 with DMXAA provides enhanced activity against the MDAH-MCa-4 tumour. It is presumed that these interactions result primarily from induction of hypoxia after DMXAA treatment, although it has been shown with melphalan that decreased extracellular pH and entrapment of the alkylating agent as a result of falling blood flow also contribute to the increased anti-tumour activity (Pruijn et al, 1997). Inhibition of tumour blood flow after DMXAA appears to be essentially irreversible (Figure 2), as noted previously (Cliffe et al, 1994), in which case it might be expected that the cells dependent on these vessels would be fated to die as a result of ischaemic damage and that the addition of a bioreductive drug would have no further

© Cancer Research Campaign 1998 

effect. The observation that bioreductive drugs increase tumour cell killing therefore argues that there is an important (treatment-limiting) population of tumour cells that are only transiently hypoxic after DMXAA treatment and that eventually contribute to tumour regrowth.

Addition of 5-HT to DMXAA–bioreductive drug combinations provides further increases in anti-tumour activity (Figure 4 and Table 1). The kinetics of blood flow inhibition after DMXAA/5-HT combinations (early inhibition with some reversal) is different from that after DMXAA alone (Figure 2), and might provide more of the transient hypoxia that can be exploited by bioreductive drugs (Brown and Koong, 1991; Brown and Lemmon, 1991). Studies of changes in blood flow (and hypoxia) at the micro-vascular level would assist in clarifying these issues.

Although 5-HT enhances the anti-tumour activity of DMXAA–bioreductive drug combinations, in the case of TIRA or CI-1010 there is an approximately similar increase in host toxicity so that little therapeutic advantage is obtained. There is evidence that the radioprotective effect of 5-HT in mice is due to induction of normal tissue hypoxia (Bacq, 1965), which might also be responsible for the enhancement of bioreductive drug toxicity in the present study. However, not all bioreductive drugs suffer from this problem; the anti-tumour effect of the dinitrobenzamide mustard SN 23816 with DMXAA is enhanced to a greater extent than is host toxicity by co-administration of 5-HT, resulting in a significant therapeutic gain (Table 1). It is not clear why the effect on host toxicity is less for SN 23816 than for the other bioreductive drugs. The oxygen dependence of TIRA cytotoxicity is quantitatively different than for RB 6145/RSU 1069 or SN 23816, with activation of the latter nitro compounds requiring much more severe hypoxia than is the case for TIRA (Koch, 1993; Wilson et al, 1994). On this basis, both SN 23816 and CI-1010 might be expected to be less sensitive than TIRA to induction of hypoxia in normal tissues. The greater host toxicity enhancement by 5-HT for CI-1010 than for SN 23816 may indicate that different normal tissues (with different blood flow responses to 5-HT) are dose limiting for the two agents.

In conclusion, the present study demonstrates that co-administration of 5-HT with DMXAA provides marked tumour blood flow inhibition at well-tolerated doses, and that this combination is therapeutically superior to DMXAA alone as an antivascular tumour therapy in mice. Further improvement in therapeutic effect is achievable by combining DMXAA/5-HT with the bioreductive drug SN 23816, although this advantage is not obtained with the other two bioreductive drugs tested. Combination of DMXAA with 5-HT, and with appropriate bioreductive drugs, may be useful for improving the efficacy of this novel antivascular agent in clinical application.

### ACKNOWLEDGEMENTS

This study was funded by the National Cancer Institute, USA (contract NO1-CM-47019) and the Health Research Council of NZ. CJL was the recipient of a Junior Award in Health Research from the Health Research Council of NZ.

### REFERENCES

Adams GE and Stratford IJ (1994) Bioreductive drugs for cancer therapy: the search for tumor specificity. *Int J Radiat Oncol Biol Phys* **29**: 231–238

Bacq ZM (1965) *Chemical Protection Against Ionizing Radiation*. Charles C. Thomas: Springfield, IL

Baguley BC and Ching L-M (1997) Immunomodulatory actions of xanthenone anticancer drugs. *Bio Drugs* **8**: 119–127

Baguley BC, Cole G, Thomsen LL and Zhuang L (1993) Serotonin involvement in the antitumour and host effects of flavone-8-acetic acid and 5,6-demethylxanthenone-4-acetic acid. *Cancer Chemother Pharmacol* **33**: 77–81

Baguley BC, Zhuang L and Kestell P (1997) Increased plasma serotonin following treatment with flavone-8-acetic acid, 5,6-dimethylxanthenone-4-acetic acid, vinblastine and colchicine: relation to vascular effects. *Oncol Res* **9**: 55–60

Bibby MC, Phillips RM, Double JA and Pratesi G (1991) Anti-tumour activity of flavone acetic acid (NSC 347512) in mice – influence of immune status. *Br J Cancer* **63**: 57–62

Bremner JCM, Adams GE, Pearson JK, Sansom JM, Stratford IJ, Bedwell J, Bown SG, MacRobert AJ and Phillips D (1992) Increasing the effect of photodynamic therapy on the RIF-1 murine sarcoma, using the bioreductive drugs RSU1069 and RB6145. *Br J Cancer* **66**: 1070–1076

Brown JM (1993) SR 4233 (Tirapazamine): a new anticancer drug exploiting hypoxia in solid tumours. *Br J Cancer* **67**: 1163–1170

Brown JM and Koong A (1991) Therapeutic advantage of hypoxic cells in tumors: a theoretical study. *J Natl Cancer Inst* **83**: 178–185

Brown JM and Lemmon MJ (1991) Tumor hypoxia can be exploited to preferentially sensitize tumors to fractionated irradiation. *Int J Radiat Oncol Biol Phys* **20**: 457–461

Brown SL, Hunt JW and Hill RP (1988) A comparison of the rate of clearance of xenon ($^{133}$Xe) and pertechnetate ion ($^{99m}$Tc $O^-_4$) in murine tumors and normal leg muscles. *Nucl Med Biol* **15**: 381–390

Chaplin DJ (1986) Potentiation of RSU-1069 tumour cytotoxicity by 5-hydroxytryptamine (5-HT). *Br J Cancer* **54**: 727–731

Ching L-M, Joseph WR and Baguley BC (1992) Antitumour responses to flavone-8-acetic acid and 5,6-dimethylxanthenone-4-acetic acid in immune deficient mice. *Br J Cancer* **66**: 128–130

Ching L-M, Joseph WR, Crosier KE and Baguley BC (1994) Induction of tumor necrosis factor-α messenger RNA in human and murine cells by the flavone acetic acid analogue 5,6-dimethylxanthenone-4-acetic acid (NSC 640488). *Cancer Res* **54**: 870–872

Cliffe S, Taylor ML, Rutland M, Baguley B, Hill RP and Wilson WR (1994) Combining bioreductive drugs (SR 4233 or SN 23862) with the vasoactive agents flavone acetic acid or 5,6-dimethylxanthenone acetic acid. *Int J Radiat Oncol Biol Phys* **29**: 373–377

Dark GG, Hill SA, Prise VE, Tozer GM, Pettit GR and Chaplin DJ (1997) Combretastatin A-4, an agent that displays potent and selective toxicity toward tumor vasculature. *Cancer Res* **57**: 1829–1834

Edwards HS, Bremner JCM and Stratford IJ (1991) Induction of tumour hypoxia by FAA and TNF: interaction with bioreductive drugs. *Int J Radiat Biol* **60**: 373–377

Finlay GJ, Smith GP, Fray LM and Baguley BC (1988) Effect of flavone acetic acid on Lewis lung carcinoma: evidence for an indirect effect. *J Natl Cancer Inst* **80**: 241–245

Futami H, Eader LA, Komschlies KL, Bull R, Gruys E, Ortaldo JR, Young HA and Wiltrout RH (1991) Flavone acetic acid directly induces expression of cytokine genes in mouse splenic leukocytes but not in human peripheral blood leukocytes. *Cancer Res* **51**: 6596–6602

Hill S, Williams KB and Denekamp J (1989) Vascular collapse after flavone acetic acid: a possible mechanism of its anti-tumour action. *Eur J Cancer Clin Oncol* **25**: 1419–1424

Hill SA, Williams KB and Denekamp J (1992) A comparison of vascular-mediated cell death by the necrotizing agent GR63178 and flavone acetic acid. *Int J Radiat Oncol Biol Phys* **22**: 437–441

Jenkins TC, Naylor MA, O'Neill P, Threadgill MD, Cole S, Stratford IJ, Adams GE, Fielden M, Suto MJ and Stier MA (1990) Synthesis and evaluation of α[[(2-haloethyl)amino]-methyl]-2-nitro-1H-imidazole-1-ethanols as prodrugs of α-[(1-aziridinyl)methyl]-2-nitro-1H-imidazole-1-ethanol (RSU-1069) and its analogues which are radiosensitizers and bioreductively activated cytotoxins. *J Med Chem* **33**: 2603–2610

Kerr DJ and Kaye SB (1989) Flavone acetic acid – preclinical and clinical activity. *Eur J Cancer Clin Oncol* **25**: 1271–1272

Koch CJ (1993) Unusual oxygen concentration dependence of toxicity of SR 4233, a hypoxic cell toxin. *Cancer Res* **53**: 3992–3997

Laws AL, Matthew AM, Double JA and Bibby MC (1995) Preclinical in vitro and in vivo activity of 5,6-dimethylxanthenone-4-acetic acid. *Br J Cancer* **71**: 1204–1209

Mahadevan V, Malik STA, Meager A, Fiers W, Lewis GP and Hart IR (1990) Role of tumor necrosis factor in flavone acetic acid-induced tumor vasculature shutdown. *Cancer Res* **50**: 5537–5542

© Cancer Research Campaign 1998

Manda T, Nishigaki F, Mori J and Shimomura K (1988) Important role of serotonin in the antitumor effects of recombinant human tumor necrosis factor-α in mice. *Cancer Res* **48**: 4250–4255

Moselen J, Hay M, Denny WA and Wilson WR (1995) N-[2-(2-methyl-5-nitroimidazolyl)-ethyl]-4-(2-nitroimidazolyl)butanamide (NNB, NSC 639862), a bis-bioreductive agent with marked selective toxicity towards hypoxic cells. *Cancer Res* **55**: 574–580

O'Reilly SM, Rustin GJS, Farmer K, Burke M, Hill S and Denekamp J (1993) Flavone acetic acid (FAA) with recombinant interleukin-2 (rIL-2) in advanced malignant melanoma. I. Clinical and vascular studies. *Br J Cancer* **67**: 1342–1345

Palmer BD, Wilson WR, Cliffe S and Denny WA (1992) Hypoxia-selective antitumor agents. 5. Synthesis of water-soluble nitroaniline mustards with selective cytotoxicity for hypoxic mammalian cells. *J Med Chem* **35**: 3214–3222

Palmer BD, Wilson WR, Atwell GJ, Schultz D, Xu XZ and Denny WA (1994) Hypoxia-selective antitumor agents. 9. Structure-activity relationships for hypoxia-selective cytotoxicity among analogues of 5-[N,N-bis(2-chloroethyl)amino]-2,4-dinitrobenzamide. *J Med Chem* **37**: 2175–2184

Pedley RB, Begent RHJ, Boden JA, Boxer GM, Boden R and Keep PA (1994) Enhancement of radioimmunotherapy by drugs modifying tumour blood flow in a colonic xenograft model. *Int J Cancer* **57**: 830–835

Pedley RB, Boden JA, Boden R, Boxer GM, Flynn AA, Keep PA and Begent RHJ (1996) Ablation of colorectal xenografts with combined radioimmunotherapy and tumor blood flow-modifying agents. *Cancer Res* **56**: 3293–3300

Peters CE and Chaplin DJ (1992) Blood flow modification in the SCCVII tumor: effects of 5-hydroxytryptamine, hydralazine, and propranolol. *Int J Radiat Oncol Biol Phys* **22**: 463–465

Philpott M, Joseph WR, Crosier KE, Baguley BC and Ching L-M (1997) Production of tumour necrosis factor-alpha by cultured human peripheral blood leucocytes in response to the antitumour agent 5,6-dimethylxanthenone-4-acetic acid (NSC 640488). *Br J Cancer* **76**: 1586–1591

Pratesi G, Rodolfo M, Rovetta G and Parmiani G (1990) Role of T cells and tumour necrosis factor in antitumour activity and toxicity of flavone acetic acid. *Eur J Cancer Clin Oncol* **26**: 1079–1083

Pruijn FB, van Daalen M, Holford NHG and Wilson WR (1997) Mechanisms of enhancement of the antitumour activity of melphalan by the tumour blood flow inhibitor 5,6-dimethylxanthenone-4-acetic acid. *Cancer Chemother Pharmacol* **39**: 541–546

Rewcastle GW, Kestell P, Baguley BC and Denny WA (1990) Light-induced breakdown of flavone acetic acid and xanthenone analogues in solution. *J Natl Cancer Inst* **82**: 528–529

Rewcastle GW, Atwell GJ, Zhuang L, Baguley BC and Denny WA (1991) Potential antitumor agents. 61. Structure-activity relationships for in vivo colon-38 activity among disubstituted 9-oxo-9H-xanthene-4-acetic acids. *J Med Chem* **34**: 217–222

Sekida T, Oyama M, Matsugi W, Matsui T, Harada K, Kotera Y and Ohashi M (1997) A novel antitumor agent: Mode of action of tumor blood flow inhibitor, KB-R8498 (abstract). *Proc Am Assoc Cancer Res* **38**: 218

Silobrcic V and Suit HD (1967) Tumor-specific antigen(s) in a spontaneous mammary carcinoma of C3H mice. I. Quantitative cell transplants into mammary-tumor-agent-positive and -free mice. *J Natl Cancer Inst* **39**: 1113–1119

Stucker O, Vicaut E and Teisseire B (1991) Hyper-responsiveness to 5-$HT_2$ agonists by tumour-linked arterioles in mice: consequences for tumour growth. *Int J Radiat Biol* **60**: 237–241

Sun J and Brown JM (1989) Enhancement of the antitumor effect of flavone acetic acid by the bioreductive cytotoxic drug SR 4233 in a murine carcinoma. *Cancer Res* **49**: 5664–5670

Vincent PW, Roberts BJ, Elliot WL and Leopold WR (1997) Chemotherapy with DMXAA (5,6-dimethylxanthenone-4-acetic acid) in combination with CI-1010 (1H-imidazole-1-ethanol,alpha-[[(2-bromoethyl)amino]methyl]-2-nitro-, mono hydrobromide (R isomer)) against advanced stage murine colon carcinoma 26. *Oncol Rep* **4**: 143–147

Wilson WR and Pruijn FB (1995) Hypoxia-activated prodrugs as antitumour agents: strategies for maximizing tumor cell killing. *Clin Exper Pharmacol Physiol* **22**: 881–885

Wilson WR, Siim BG, Moselen JM and Pullen SM (1994). Quantitative oxygen dependence (K values) for bioreductive drug cytotoxicity (abstract). *Proc Am Assoc Cancer Res* **35**: 364

Wilson WR, Denny WA, Pullen SM, Thompson KM, Li AE, Patterson LH and Lee HH (1996) Tertiary amine N-oxides as bioreductive drugs: DACA *N*-oxide, nitracrine *N*-oxide and AQ4N. *Br J Cancer* **74**: (suppl. XXVII): S43–S47

Zwi LJ, Baguley BC, Gavin JB and Wilson WR (1989) Blood flow failure as a major determinant in the antitumor action of flavone acetic acid. *J Natl Cancer Inst* **81**: 1005–1013

Zwi LJ, Baguley BC, Gavin JB and Wilson WR (1990) The use of vascularised spheroids to investigate the action of flavone acetic acid on tumour blood vessels. *Br J Cancer* **62**: 231–237

Zwi LJ, Baguley BC, Gavin JB and Wilson WR (1994*a*) Correlation between immune and vascular activities of xanthenone acetic acid antitumor agents. *Oncol Res* **6**: 79–85

Zwi LJ, Baguley BC, Gavin JB and Wilson WR (1994*b*) The morphological effects of the anti-tumour agents flavone acetic acid and 5,6-dimethylxanthenone acetic acid on the colon 38 mouse tumour. *Pathology* **26**: 161–169

© Cancer Research Campaign 1998